# Genomics of the critically endangered monotypic genus Sinopora: the plastome of *S. hongkongensis* (Lauraceae)

**DOI:** 10.1080/23802359.2019.1703590

**Published:** 2020-01-07

**Authors:** Angelo Damian Armijos Carrion, Damien Daniel Hinsinger, Joeri Sergej Strijk

**Affiliations:** a Biodiversity Genomics Team, Plant Ecophysiology and Evolution Group, Guangxi Key Laboratory of Forest Ecology and Conservation, College of Forestry, Guangxi University, Nanning, China;; b Alliance for Conservation Tree Genomics, Pha Tad Ke Botanical Garden, Luang Prabang, Laos;; c State Key Laboratory for Conservation and Utilization of Subtropical Agro-Bioresources, College of Forestry, Guangxi University, Nanning, China

**Keywords:** Lauraceae, complete chloroplast genome, Critically Endangered, *Sinopora hongkongensis*, genomics

## Abstract

*Sinopora hongkongensis* is a critically endangered endemic tree species restricted to Hong Kong. Here we report its plastome sequence. The *S. hongkongensis* plastome was 158,612 bp in length, with a large single-copy (LSC) region of 89,405 bp and a small single-copy (SSC) region of 18,205 bp, separated by two inverted repeat (IR) regions of 25,498 bp. It contained 126 genes, including 89 coding genes, 29 tRNA genes, and 8 rRNA genes. The overall GC content was 39.0%, and 43.0%, 37.7%, and 34.0%, in the IRs, LSC, and SSC regions, respectively. A phylogenetic analysis combining a subset of Lauraceae plastomes with closely related outgroup families confirms the placement of *S. hongkongensis* in Lauraceae and explores relationships with other genera in the family.

Lauraceae are a major plant family in Magnoliids, consisting of more than 55 genera distributed worldwide (Oliveira-Filho et al. 2015; Zhao et al. 2018). Its species are distributed mainly in subtropical or tropical warm temperate regions consisting of woody elements ranging from small treelets to large canopy elements (Lim 2012). Species in the family form an important ecological and economic component of Asian forests, providing valuable sources of medicine, timber, spices, nutritious fruits, and perfumes. In China, Lauraceae are represented by more than 400 species from nearly all Asian genera (25; sensu Li, Li, et al. 2008), in addition to two monotypic endemic Chinese genera (*Sinopora* J. Li et al. and *Sinosassafras* H. W. Li). Despite the importance of the family, the evolutionary relationships in Lauraceae are still poorly understood (Rohwer 2000; Huang et al. 2016; Liu et al. 2017). Availability of genomic resources is steadily increasing in the family (e.g. Liang et al. 2019; Song et al. 2019), but for most genera, such data are still lacking, hindering a clear evaluation of their taxonomic and evolutionary status (Rohwer et al. 2014; Hinsinger and Strijk 2017a; Zhao et al. 2018).

*Sinopora hongkongensis* (Xia et al. 2006; Li, Xia, et al. 2008), the only species in the genus *Sinopora*, is restricted to the evergreen broad-leaved forests of Hong Kong. The species is here classified under the IUCN Red List Assessment criteria as Critically Endangered B1 + 2ab,C2a(i),D. Although the habitat in which the last remaining individuals are found is under protective legislation (and exact locality details are restricted), the species is balancing on the brink of extinction without the development of ex-situ approaches such as micropropagation.

Here, we report the plastome sequence of *S. hongkongensis* to provide resources in support of further genomic and conservation genetic studies on this exceptionally rare species.

Supported by staff from the Agriculture, Fisheries and Conservation Department (AFCD, HK), we visited one individual of *S. hongkongensis* and collected leaves which were field preserved in dry ice. We extracted genomic DNA as described before (Cvetković et al. 2019), from 0.1 g of frozen fresh leaves. Voucher material was deposited at the herbarium of the Agriculture, Fisheries and Conservation Department (AFCD, HK) and the Biodiversity Genomics Team tissue bank (BGT; Strijk 3723). Library construction and sequencing were performed by Novogene (Beijing, China) using the NEBNext Ultra II DNA Library Prep Kit (Ipswich, Massachusetts, USA), as described elsewhere (Hinsinger and Strijk 2017a, 2017b, 2017c). *De novo* assembly of the cp genome was conducted using org.asm v0.2.05 (ORG.ASM 2016) and annotated using cpGAVAS (Liu et al. 2012).

The plastome of *S. hongkongensis* (GenBank accession number MN722652) was 158,612 bp in length, with a large single-copy (LSC) region of 89,405 bp and a small single-copy (SSC) region of 18,205 bp, separated by two inverted repeat regions (IRs) of 25,498 bp. The genome contained 126 genes, including 89 coding genes, 29 tRNA genes, and 8 rRNA genes. The overall GC content was 39.0%, and 43.0%, 37.7%, 34.0%, in the IRs, LSC, and SSC regions, respectively.

We reconstructed a maximum-likelihood phylogenetic tree including representative species in Lauraceae and other families using RaxML-NG v0.8.1 (Kozlov et al. 2019), with 1000 bootstrap replicates and the GTR + I+G4 substitution model, as selected by ModelTest-NG v0.1.5 (Darriba et al. 2019). This phylogenetic analysis indicated that *S. hongkongensis* is sister to *Beilschmiedia pauciflora* (Figure 1). All nodes in the plastome ML trees were strongly supported. The complete plastome sequence of *S. hongkongensis* will provide a useful resource for its conservation as well as future phylogenetic studies in this genus.

**Figure 1.**
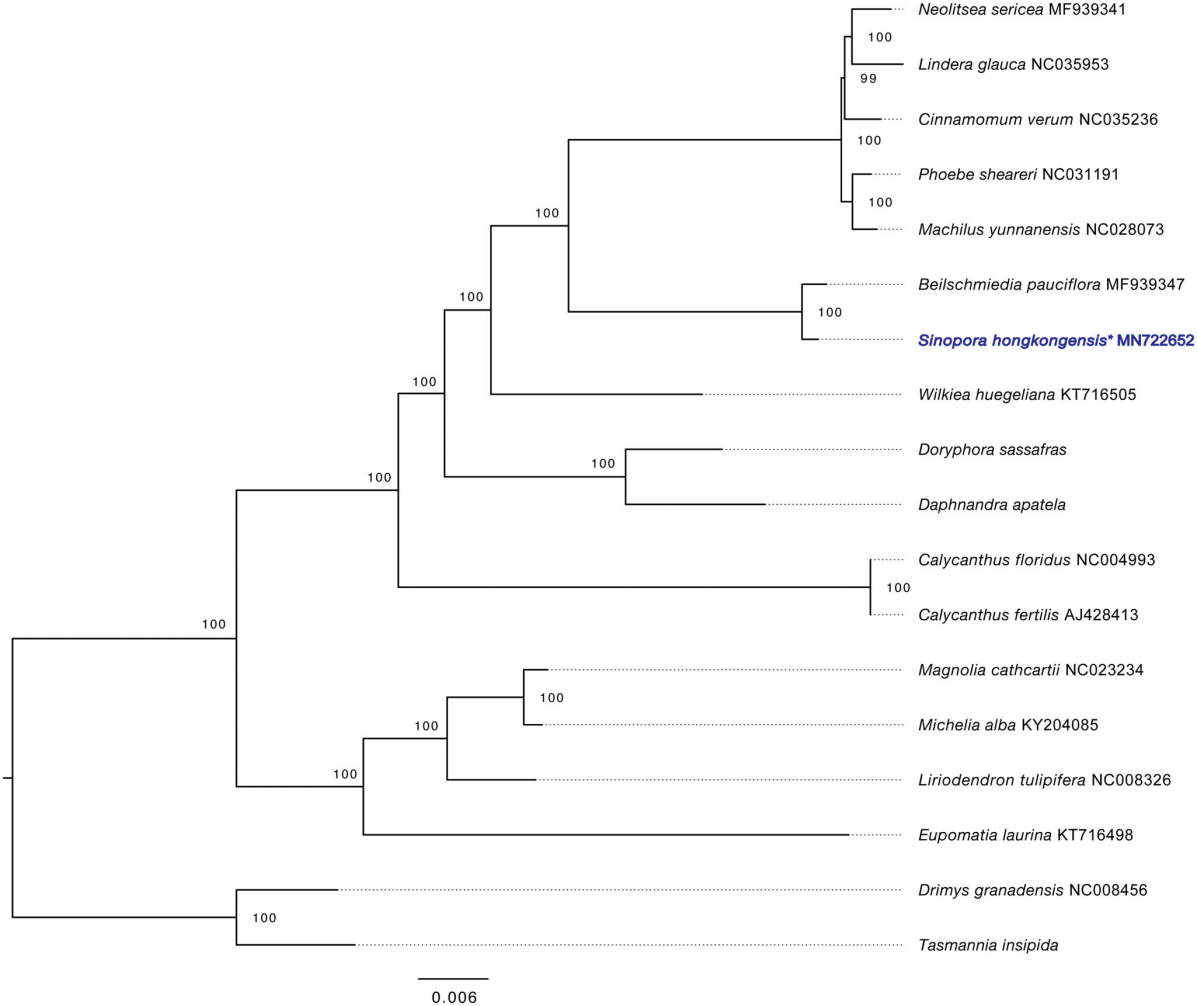
ML phylogenetic tree based on plastome data showing relationships between representative genera in Lauraceae and selected outgroup taxa. The position of *S. hongkongensis* is highlighted with an asterisk. Bootstrap support values (1000 replicates) are indicated on branches. Plastomes of *Doryphora sassafras*, *Daphnandra apatela*, and *Tasmannia insipida* were obtained from Dryad (doi.org/10.5061/dryad.j64j0).

## References

[CIT0001] Cvetković T, Hinsinger DD, Strijk JS. 2019. Exploring evolution and diversity of Chinese Dipterocarpaceae using next-generation sequencing. Sci Rep. 9(1):11639. 31406227 10.1038/s41598-019-48240-y PMC6690942

[CIT0002] Darriba D, Posada D, Kozlov AM, Stamatakis A, Morel B, Flouri T. 2019. ModelTest-NG: a new and scalable tool for the selection of DNA and protein evolutionary models. BioRxiv. 612903. 10.1093/molbev/msz189 PMC6984357 31432070

[CIT0003] Hinsinger DD, Strijk JS. 2017a. Toward phylogenomics of Lauraceae: the complete chloroplast genome sequence of *Litsea glutinosa* (Lauraceae), an invasive tree species on Indian and Pacific Ocean islands. Plant Gene. 9:71–79.

[CIT0004] Hinsinger DD, Strijk JS. 2017b. Complete chloroplast genome sequence of *Castanopsis concinna* (Fagaceae), a threatened species from Hong Kong and South-Eastern China. Mitochondrial DNA A DNA Mapp Seq Anal. 28(1):65–66. 26678387 10.3109/19401736.2015.1110800

[CIT0005] Hinsinger DD, Strijk JS. 2017c. The chloroplast genome sequence of *Michelia alba* (Magnoliaceae), an ornamental tree species. Mitochondrial DNA B. 2(1):9–10. 10.1080/23802359.2016.1275850 PMC7800174 33473697

[CIT0006] Huang J-F, Li L, Conran JG, Li J. 2016. Phylogenetic utility of LEAFY gene in Cinnamomum (Lauraceae): gene duplication and polymerase chain reaction-mediated recombination. J Syst Evol. 54(3):238–249.

[CIT0007] Kozlov AM, Darriba D, Flouri T, Morel B, Stamatakis A. 2019. RAxML-NG: a fast, scalable and user-friendly tool for maximum likelihood phylogenetic inference. Bioinformatics. 35(21):4453–4455. 31070718 10.1093/bioinformatics/btz305 PMC6821337

[CIT0008] Li HW, Li J, Huang PH, Wei FN, Cui HB, van der Werff H. 2008. Lauraceae. In: Wu ZY, Raven PH, Hong DY, editors. Flora of China. Vol. 7. Beijing: Science Press; St. Louis: Missouri Botanical Garden Press; p. 102–254.

[CIT0009] Li J, Xia N.-H, Li X.-W. 2008. Sinopora, a new genus of Lauraceae from South China. Novon. 18(2):199–201.

[CIT0010] Liang X-J, Hinsinger DD, Elias RB, Strijk JS. 2019. The plastome sequence of *Laurus azorica* (Seub.) Franco, an endemic tree species of the Azores islands. Mitochondrial DNA B. 4(1):363–365.

[CIT0011] Lim TK. 2012. Edible medicinal and non-medicinal plants. Vol. 1. Dordrecht: Springer; p. 285–292.

[CIT0012] Liu C, Shi L, Zhu Y, Chen H, Zhang J, Lin X, Guan X. 2012. CpGAVAS, an integrated web server for the annotation, visualization, analysis, and GenBank submission of completely sequenced chloroplast genome sequences. BMC Genomics. 13:715. 23256920 10.1186/1471-2164-13-715 PMC3543216

[CIT0013] Liu ZF, Ci XQ, Li L, Li HW, Conran JG, Li J. 2017. DNA barcoding evaluation and implications for phylogenetic relationships in Lauraceae from China. PLoS One. 12(4):e0175788. 28414813 10.1371/journal.pone.0175788 PMC5393608

[CIT0014] Oliveira-Filho AA, Fernandes HM, Assis TJ. 2015. Lauraceae’s family: a brief review of cardiovascular effects. Int J Pharmacogn Phytochem Res. 7:22–26.

[CIT0015] ORG.ASM. 2016. The ORGanelle ASeMbler. http://pythonhosted.org/ORG.asm/index.html#

[CIT0017] Rohwer JG. 2000. Toward a phylogenetic classification of the Lauraceae: evidence from matK sequences. Syst Bot. 25(1):60–71.

[CIT0018] Rohwer JG, de Moraes PLR, Rudolph B, Van der Werff H. 2014. A phylogenetic analysis of the Cryptocarya group (Lauraceae), and relationships of Dahlgrenodendron, Sinopora, Triadodaphne, and Yasunia. Phytotaxa. 158(2):111–132.

[CIT0019] Song Y, Yu WB, Tan YH, Jin JJ, Wang B, Yang JB, Liu B, Corlett RT. 2019. Plastid phylogenmics improve phylogenetic resolution in the Lauraceae. J Syst Evol. Vol.0, (00):1–17.

[CIT0020] Xia NH, Deng YF, Yip KL. 2006. Syndiclis hongkongensis (Lauraceae), a new species from. China J Trop Subtrop Bot. 14:75–77.

[CIT0022] Zhao M-L, Song Y, Ni J, Yao X, Tan Y-H, Xu Z-F. 2018. Comparative chloroplast genomics and phylogenetics of nine Lindera species (Lauraceae). Sci Rep. 8(1):8844. 29891996 10.1038/s41598-018-27090-0 PMC5995902

